# Cutting-edge nanotechnology: unveiling the role of zinc oxide nanoparticles in combating deadly gastrointestinal tumors

**DOI:** 10.3389/fbioe.2025.1547757

**Published:** 2025-03-20

**Authors:** Yonggang Guo, Mohammadamin Morshedi

**Affiliations:** ^1^ Pingdingshan College, Pingdingshan, Henan, China; ^2^ School of Medicine, Kashan University of Medical Sciences, Kashan, Iran

**Keywords:** gastrointestinal cancer, nanotechnology, ZnO, therapy, nanoparticles

## Abstract

Zinc oxide nanoparticles (ZnO-NPs) have gained significant attention in cancer therapy due to their unique physical and chemical properties, particularly in treating gastrointestinal (GI) cancers such as gastric, colorectal, and hepatocellular carcinoma. These nanoparticles generate reactive oxygen species (ROS) upon entering cancer cells, causing oxidative stress that leads to cellular damage, DNA fragmentation, and apoptosis. ZnO-NPs affect the expression of key proteins involved in apoptosis, including p53, Bax, and Bcl-2, which regulate cell cycle arrest and programmed cell death. Additionally, ZnO-NPs can reduce mitochondrial membrane potential, further enhancing apoptosis in cancer cells. Furthermore, ZnO-NPs inhibit cancer cell proliferation by interfering with cell cycle progression. They reduce levels of cyclins and cyclin-dependent kinases (CDKs), leading to cell cycle arrest. ZnO-NPs also exhibit anti-metastatic properties by inhibiting the migration and invasion of cancer cells through modulation of signaling pathways that affect cell adhesion and cytoskeletal dynamics. The efficacy of ZnO-NPs in overcoming chemotherapy resistance has been demonstrated by their ability to reduce the IC50 values of chemotherapeutic agents, making cancer cells more susceptible to drug-induced cell death. In this review, we summarize the mechanisms by which ZnO-NPs exert anticancer effects in GI cancers, focusing on apoptosis, cell cycle regulation, and metastasis inhibition, while also highlighting the current limitations in translating these findings into effective clinical treatments.

## Introduction

Nanotechnology is a branch of scientific exploration that integrates concepts from multiple disciplines such as chemistry, engineering, biology, and medicine (Adir et al., 2020; Wang Q. et al., 2022; Kanaoujiya et al., 2022). There are a number of advantageous uses of nanotechnology in cancer research, which include identifying tumors and cancer biomarkers at an early stage and developing treatment plans that are not achievable with traditional methods. Conducting a thorough examination of the management and detection of digestive tract cancers is crucial in decreasing the prevalence and mortality rates of the disease and increasing the likelihood of patient recovery (Dalhammar et al., 2020; Piazuelo and Correa, 2013; Yu et al., 2019). The implementation of nanotechnology in the field of cancer research has sparked hope among scientists for the development of groundbreaking methods for treating cancer (Gao et al., 2021). Much effort has been dedicated to developing innovative approaches for diagnosing and treating gastrointestinal (GI) cancers, with the goal of enhancing the overall health and prolonging the lives of individuals as such cancers account for over 50% of all cancer-related fatalities (Santoni et al., 2022; Stathis and Moore, 2010).

Zinc oxide nanoparticles (ZnO NPs) have been increasingly recognized in recent years for their potential in treating cancer (Zhang et al., 2011). Various studies have suggested that ZnO NPs possess the ability to induce harm in cells in a distinct manner, which varies for each cell type and is reliant on their pace of proliferation. Cells that rapidly multiply, like cancer cells, appear to be more susceptible to damage, whereas inactive cells show the least amount of susceptibility (Hanley et al., 2008; Ostrovsky et al., 2009; Premanathan et al., 2011). Based on the available evidence, the specific kind of cell being examined significantly impacts the potential harmful effects exerted by ZnO NPs on mammalian cells. Currently, there is insufficient knowledge about the full extent of ZnO nanoparticles’ ability to combat cancer and the precise mechanisms by which they induce apoptosis in cancerous cells. Our evaluation focuses on the potential of ZnO NPs to treat gastrointestinal cancer by summarizing noteworthy discoveries from recent studies and examining the proposed mechanisms that contribute to their targeted harmful effects on GI cancer cells.

### Zinc oxide nanoparticles and cancer therapy

ZnO NPs are becoming increasingly popular in the field of research on nanoparticles owing to their unique physical and chemical properties, making them a desirable choice for drug delivery, cancer detection, and treatment. ZnO NPs have been proven to be extremely efficient in combating a wide range of diseases, not limited to just cancer. Furthermore, ZnO NPs have proven to have beneficial applications in fields such as beauty, technology, and clothing manufacturing (Smijs and Pavel, 2011; Ruszkiewicz et al., 2017; Vidor et al., 2017; Vasantharaj et al., 2021). There are numerous approaches for synthesizing ZnO NPs, including chemical, physical, and biological methods. These methods require a substantial amount of energy and involve the use of high pressure or temperature during synthesis. Examples of chemical techniques include precipitation, microemulsion, chemical reduction, sol–gel, and hydrothermal processes (Brintha and Ajitha, 2015; Król et al., 2017; Li et al., 2009; Haq et al., 2017). There is a potential to synthesize ZnO NPs through physical methods such as vapor deposition, plasma treatment, and ultrasonic irradiation, though these are not as commonly employed as chemical methods (Król et al., 2017; Dong et al., 1997; Huang et al., 2001). These methods typically necessitate a considerable expenditure of energy and use of large equipment, leading to higher expenses for the goods. A technique used to acquire ZnO NPs involves using biological methods, which has become a more sustainable approach due to its minimal impact on the environment (Bandeira et al., 2020). Regardless of the technique utilized, all forms of ZnO NPs have demonstrated effectiveness in combating cancer through their ability to diagnose, treat, and consistently and specifically release anticancer medications (Akhtar et al., 2012; Chung et al., 2015; Abbasi B. A. et al., 2019).

**FIGURE 1:**
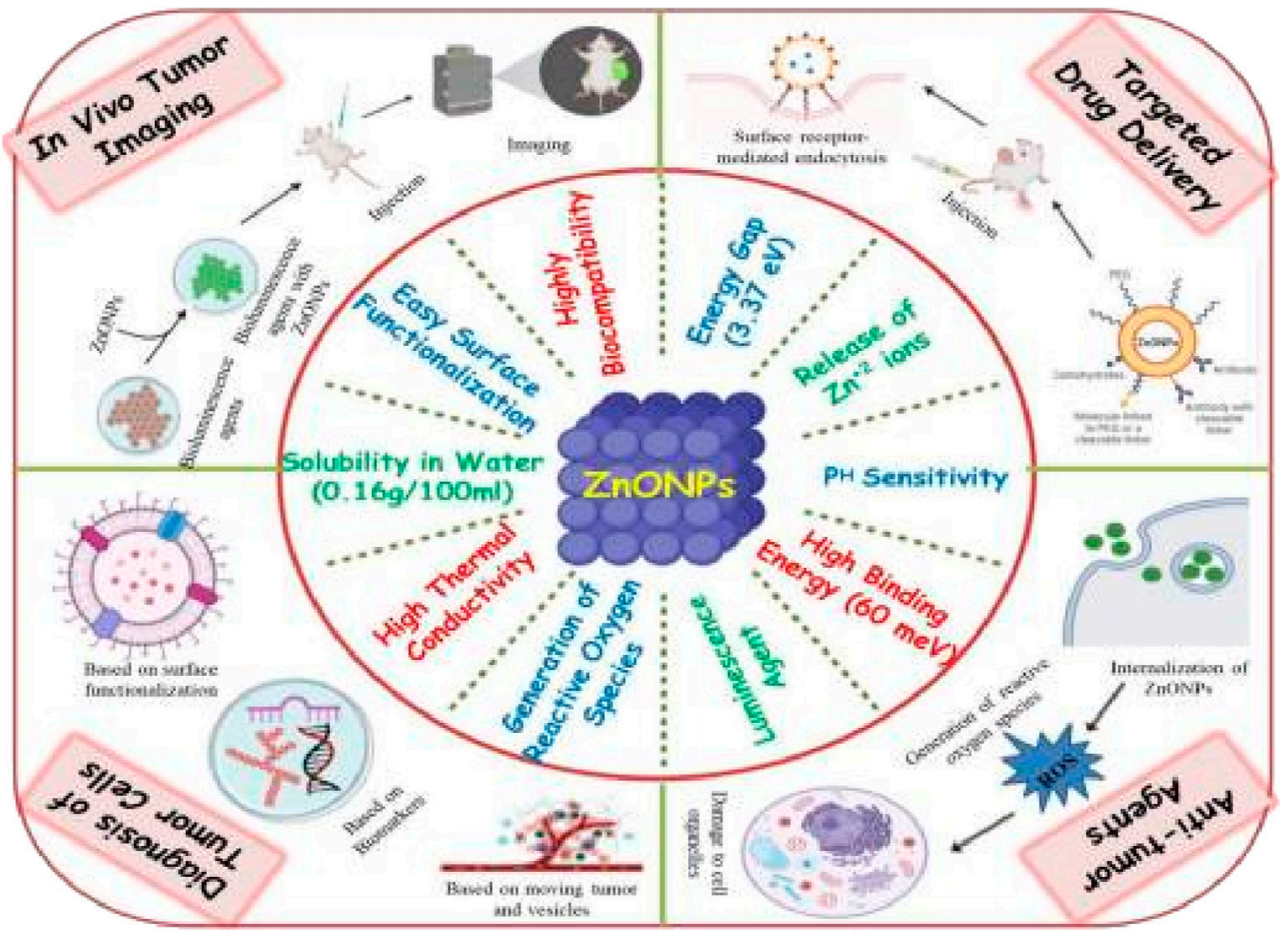
A visual depiction showing the distinct characteristics of ZnO nanoparticles and their diverse uses in combating cancer.

ZnO NPs are extensively used in numerous industries and research facilities due to their considerable range of uses (Akintelu and Folorunso, 2020). The human body is able to easily absorb zinc from nano-sized zinc oxide particles as a result of its incredibly tiny dimensions. These nanoparticles, which are relatively inexpensive and less harmful than other metal oxide nanoparticles, offer a wide range of medicinal uses, including combating bacterial infections, treating diabetes, reducing swelling, delaying the natural aging process, assisting with wound healing, and supporting bio-imaging methods (Mishra et al., 2017; Zhang and Xiong, 2015; Kim et al., 2017; Xiong, 2013). ZnO NPs are highly compatible with the human body, making them ideal for use in medical treatments as they can effectively fight against bacteria, fungi, viruses, and cancer cells (Wiesmann et al., 2020). Numerous varieties of non-organic metal compounds, including titanium dioxide, copper oxide, and zinc oxide, have been developed and are currently being studied. Out of all these metal oxides, ZnO NPs are particularly intriguing due to their cost-effectiveness, safety, and ease of production (Jayaseelan et al., 2012). In addition, any agent designed for human consumption to treat different illnesses must possess the following traits: the product must be non-toxic and free from any interaction with both food and its packaging. Additionally, it should have a pleasing flavor or be entirely tasteless, and it must not give off any unpleasant odors. A specific variety of non-organic metal compounds that meet all of the stipulated criteria is ZnO NPs. Therefore, they can be employed without causing any harm as a remedial agent, wrapping preservative, and as an anti-bacterial agent (Baum et al., 2000; Hiller and Perlmutter, 1971). Thus, the Food and Drug Administration (FDA) has classified ZnO NPs as a safe substance, known as “GRAS” (generally regarded as safe), in the United States (Rasmussen et al., 2010). Because of their large energy difference of 3.37 eV and strong attraction between particles of 60 meV, ZnO NPs possess many properties characteristic of semiconductors, including a powerful ability to speed up chemical reactions, effective light manipulation, ability to block UV light, anti-swelling effects, and potential to promote wound healing (Mirzaei and Darroudi, 2017). They have also been widely employed in cosmetics such as sun creams due to their potential to block out UV rays (Newman et al., 2009). In the rubber industry, ZnO NPs were initially used to enhance the durability of rubber composites, strengthen high polymers, and offer anti-aging benefits (Kołodziejczak-Radzimska and Jesionowski, 2014). The use of ZnO nanoparticles has become a notable area of focus in the field of biomedical imaging because of their ability to display luminescence (Shukla and Iravani, 2018). The creation of diagnostic tools has caught the attention of researchers, who are interested in using them for biosensing purposes (Barui et al., 2018). The distinct qualities of ZnO NPs, which enable them to serve as strong anti-cancer agents, are outlined in Figure 1.

### Zinc oxide nanoparticle synthesis methods

By altering the parameters of the synthesis process, a diverse range of methods can be used to synthesize ZnO nanoparticles. The method chosen for producing zinc oxide particles is greatly influenced by the intended use of the particles as there are several methods that can produce particles in different shapes and sizes (Wojnarowicz et al., 2020; Noman et al., 2022; Thakral et al., 2021; Rohani et al., 2022). Multiple approaches for producing ZnO-NPs are outlined below.

#### Hydrothermal method

This technique entails mixing precursor chemicals in a liquid solution and exposing them to elevated levels of pressure and heat, which initiates a reaction between the chemicals, resulting in the formation of nanoparticles. The method’s low temperature requirements, environmentally friendly nature, affordability, ability to be scaled, use of common equipment, and ease of application make it a highly appealing option as a substitute for other synthesis techniques. By changing the heat, time, and amount of the raw material used in the hydrothermal method, the form and dimensions of the particles can be modified. The primary process involved producing solutions of dehydrated zinc acetate (Zn(CH_3_COO)2(H_2_O)2), mixing NaOH with wood alcohol, and slowly adding it to the solution of (CH_3_COO)2⋅2H_2_O while continuously stirring. The pH level was then adjusted to range between 8 and 11. The suspension is gathered and subsequently transferred into a stainless-steel autoclave lined with Teflon, where it is maintained at a temperature of 120 °C for a duration of 6–12 h. The final product is rinsed using a combination of water and wood alcohol. The process of freeze-drying is carried out until the suspension is transformed into a powdered form of ZnO NPs (Zhang et al., 2022a). Several studies have concluded that ultrasonic-assisted synthesis techniques yield ZnO-NPs with smoother particle sizes and improved distribution and in combination with other particles against GI cancers (Wojnarowicz et al., 2018a; Liu et al., 2023).

#### Precipitation method

This method involves mixing a starting solution that includes zinc salts with a suitable substance. Under specific circumstances, this substance may serve as either a base or an acid and ultimately produce ZnO nanoparticles. Zinc nitrate and urea have the potential to be used as base materials in chemical procedures that generate ZnO-NPs, while being subjected to high temperatures and pressure within a tightly sealed water-based environment. The effectiveness of the precipitation method and its unique benefits, including its quickness, cost-effectiveness, and simplicity, were carefully considered. The simultaneous occurrence of nucleation and growth during the ZnO growth process presented challenges in the comprehensive study of the process. The basic process involves combining urea with distilled water and stirring it for a duration of 30 min. This leads to the formation of a solution of urea that is then utilized to precipitate the intended substance. Following a rigorous 2-h swirling process at 70°C, the solution with zinc nitrate underwent a total transformation and assumed a cloudy white appearance. Moreover, the previously mentioned white precursor material was centrifuged at a rate of 8000 rotations per minute for 10 min and was subsequently washed with distilled water, aiming to remove any impurities or unused ions that could have been absorbed. By using a muffle furnace, the resulting substance can be subjected to calcination for a period of 3 h at a temperature of 500°C in the presence of air (Wang et al., 2017; Adam et al., 2018; Dhivya et al., 2018).

#### Chemical vapor transport method

The process entails using a medium, such as graphite or iodine, to increase the heat of the powder and transferring it to a colder location, resulting in the formation of microscopic particles through condensation. In 1852, Bunsen was the first to introduce a method called “Chemical Vapor Transfer,” for distinguishing heterogeneous reactions with a shared characteristic. ZnO NPs are produced through a reaction between oxygen and zinc or an oxygen mixture, which occurs as the vapors are transported and interact with subsequent zones. The process of achieving the breakdown of ZnO was uncomplicated, direct, and effortless in nature. In addition, it was necessary to heat the zinc powder in the presence of oxygen, and this process had to be carefully managed to maintain a proper ratio between oxygen pressure and zinc vapor pressure so that the resulting ZnO nanostructures would be suitable. Modifications in this proportion resulted in notable discrepancies in the physical structure of ZnO, specifically in the dimensions and shape of the nanostructures (Xue et al., 2015; Colibaba, 2019; Güell et al., 2021). Studies have used this technique for the development of different ZnO nanostructures for the diagnosis and treatment of GI cancer cells (Ortiz-Casas et al., 2021).

### Biological/green synthesis methods

A superior and unique means of producing nanoparticles, as opposed to chemical and physical methods, is through the biological process, commonly referred to as “eco-friendly” manufacturing. ZnO was predominantly used as a component in food, a dietary supplement, and a constituent in pharmaceuticals. Instead of using dangerous substances, the incorporation of harmless reagents like water and organic extracts proved to be a remarkable method for producing metal nanoparticles (Barani et al., 2021; Sana et al., 2021; Maťátková et al., 2022). The significant advancements in nanobiotechnology have resulted in a multitude of potential uses for ZnO-NPs produced through biotechnological techniques in the medical field. Scientists have studied proteins, DNA, and different components of plants such as roots, stems, leaves, flowers, and fruits as a viable substitute for using chemical and physical methods to create ZnO-NPs in a risk-free manner. These organic compounds harness biochemical reactions and enzymatic mechanisms within small living organisms (Bandeira et al., 2020; Rajeshkumar et al., 2018; Ogunyemi et al., 2019; Zheng et al., 2015). A range of small living organisms possess essential components such as proteins, amino acids, DNA, enzymes, bacteriophages, and identifiable genes that can be used to produce ZnO NPs. In particular, DNA has the ability to act as a guide in ZnO-NP formation and impact their growth process. Moreover, the immense potential of the use of biotechnology techniques for producing ZnO-NPs offers a wide range of possibilities in the biological field. This includes applications such as labeling in labs, cell cultivation, gene and drug transportation, as well as nanomedicines (Banoee et al., 2010; Zelechowska et al., 2016; Iravani and Zolfaghari, 2022).

#### Plant-mediated synthesis

**FIGURE 2:**
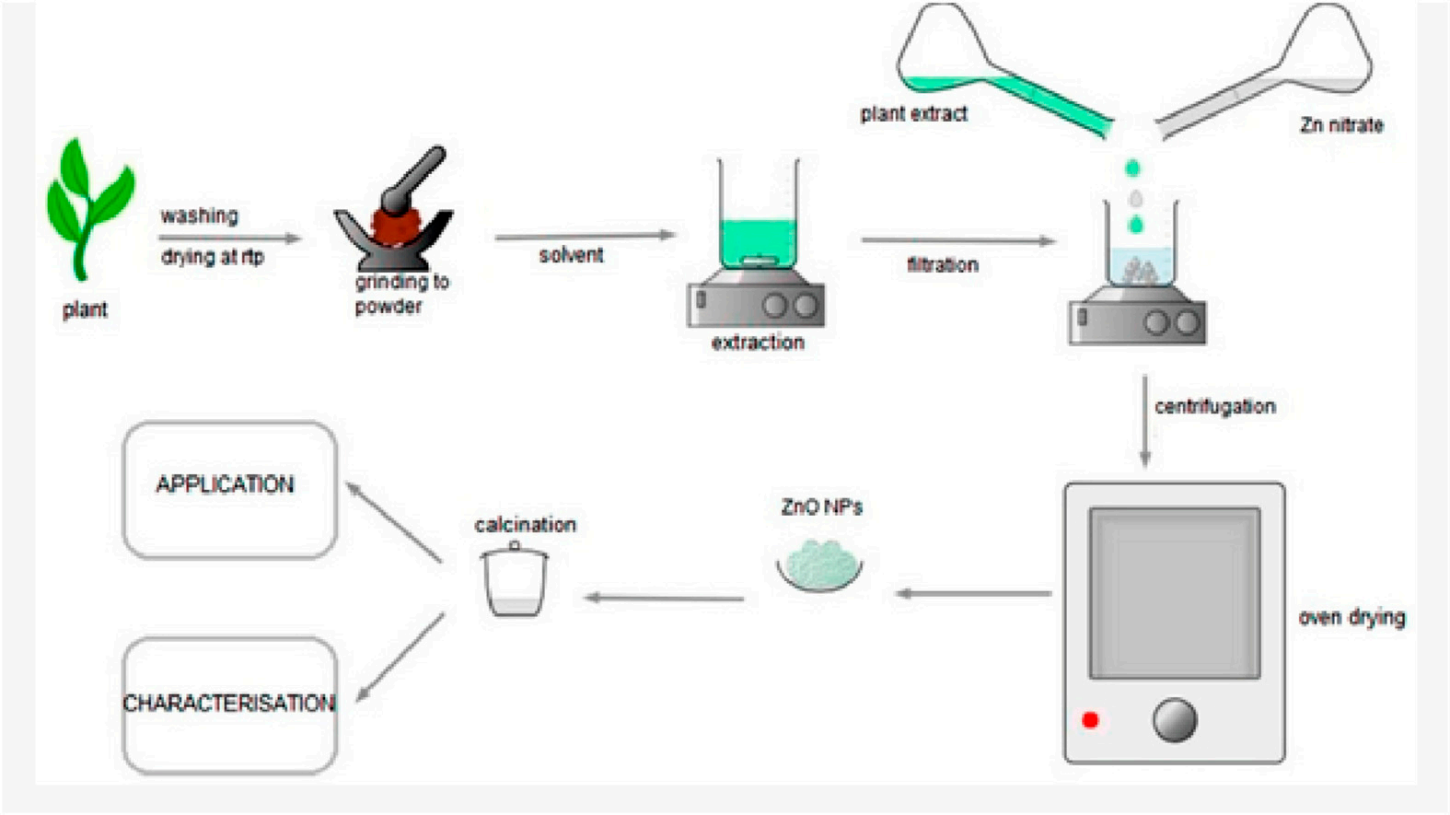
A diagram of the typical method used to synthesize ZnO nanoparticles (NPs).

An intriguing alternative to traditional methods using chemicals involves leveraging the potential of plants and plant extracts to produce ZnO-NPs (Ogunyemi et al., 2019). The incorporation of different plants and the extracts derived from them offers an extremely appealing, innovative, and reliable option. Figure 2 displays a common procedure for creating ZnO-NPs, specifically designed to prevent the use of detrimental and unsafe materials (Bao and Lan, 2019; Araya-Sibaja et al., 2022). Researchers have multiple plants and techniques available to synthesize ZnO nanoparticles. Tomato fruits, chamomile flowers, and olive plant leaves were rinsed with distilled water and then air-dried. After crushing leaves, flowers, and fruits, water was extracted from them at a temperature of 60°C–70°C for 4 h, resulting in a 200-mL solution. Afterward, the retrieved substances were cooled down to standard temperature and filtered using a piece of paper to divide the particles. The extracted substances from the plant were combined with ZnO in a separate flask, causing a chemical reaction that led to the formation of ZnO nanoparticles. The mixture was intensely stirred for a duration of 4 h, with 100 rotations per minute, while being exposed to heat. Later, it was spun at a strength equivalent to 10,000 times the force of gravity for 20 min, resulting in the separation of the top liquid portion and the collection of the solid particles. The solid that formed was rinsed with pure water and underwent a process of freeze-drying to produce ZnO NPs (Dixon et al., 2016; Paul et al., 2019). In a systematic review study, researchers showed the high efficacy of ZnO nanoparticles produced through this method against colorectal cancer cells (Mesas et al., 2023).

#### Microorganism-mediated synthesis

There has been significant attention given to using microbes, with multiple investigations carried out by using diverse types of microorganisms. Despite the challenges involved in tasks such as managing cell cultures and carrying out intracellular production and purification steps, the process of nanoparticle synthesis using microorganisms is complicated. The precise mechanism of the production of ZnO-NPs remains not fully understood (Changchun et al., 2011; Muhammad et al., 2019; Reghioua et al., 2021). Currently, the exact process by which ZnO-NPs are created is not thoroughly comprehended. The key requirements for producing ZnO NPs involve carefully choosing microorganisms, ensuring they have the ideal conditions for growth, and determining the method of biosynthesis, whether it is within or outside the cells. On a regular basis, researchers commonly employed *Sphingobacterium thalpophilum*, *Staphylococcus aureus*, and *Bacillus megaterium* NCIM2326 to produce ZnO-NPs with sizes that ranged from 10 to 95 nm. These nanoparticles were shaped into rods, cubes, a variety of forms, triangles, and needles. The nearly spherical ZnO-NPs, measuring ∼46 nm in diameter, were produced through the application of the *Lactobacillus plantarum* strain TA4 (Mohd Yusof et al., 2020). Hexagonal wurtzite and smooth/elongated ZnO NPs, measuring 10–61 nm, were produced using *Pichia kudriavzevii* and *Pichia fermentans* JA2 microorganisms. *Chlamydomonas reinhardtii* and *Sargassum muticum* have both been used without any known safety concerns. These techniques were notably known for their use of entirely natural substances and straightforward methods, making them ideal for conducting production processes with a large production volume. Abdelhakim et al. found high potential of ZnO-NPs synthesized from the culture filtrate of endophytic fungus, *A. tenuissima*, against HepG-2 cancer cell lines (Sumanth et al., 2020).

#### Arc plasma

A commonly employed technique for producing significant amounts of nanomaterials by means of condensation and evaporation is called electrical arc discharge-mediated synthesis. Islam and his team used a method in which electrical discharge was used to evaporate gas in order to produce ZnO NPs. The zinc rod served as the source of zinc, while oxygen from the air acted as the source of oxygen. A carbon rod was used as the cathode (Islam et al., 2022).

#### Thermal evaporation

A commonly employed method of creating ZnO nanostructures involves the combination of graphite and ZnO powder as a means of a reducing agent in order to attain a fully refined form of ZnO, typically at a temperature between 1000°C and 1100°C. The addition of carbon caused the reduction of the ZnO precursor, originally with a high melting point of 1975°C. As a result, the reaction led to both Zn and ZnOx being produced, with a boiling point of 500°C, which then reacted with oxygen to produce ZnO products. Thermal evaporation is capable of producing completely pure ZnO nano/microstructures through the use of different metallic elements as reducing agents (Lv et al., 2010).

#### Physical vapor deposition

The method of creating ZnO nanowires at a low temperature of 450°C was fairly uncomplicated. These nanowires were grown without the need for a catalyst, as shown by the fact that their diameter increased with an increase in temperature (Lyu et al., 2003; Eremina et al., 2022). These results showed great potential for developing nanoscale devices with ZnO on a variety of substrates that can withstand low temperatures.

#### Ultrasonic irradiation

**FIGURE 3:**
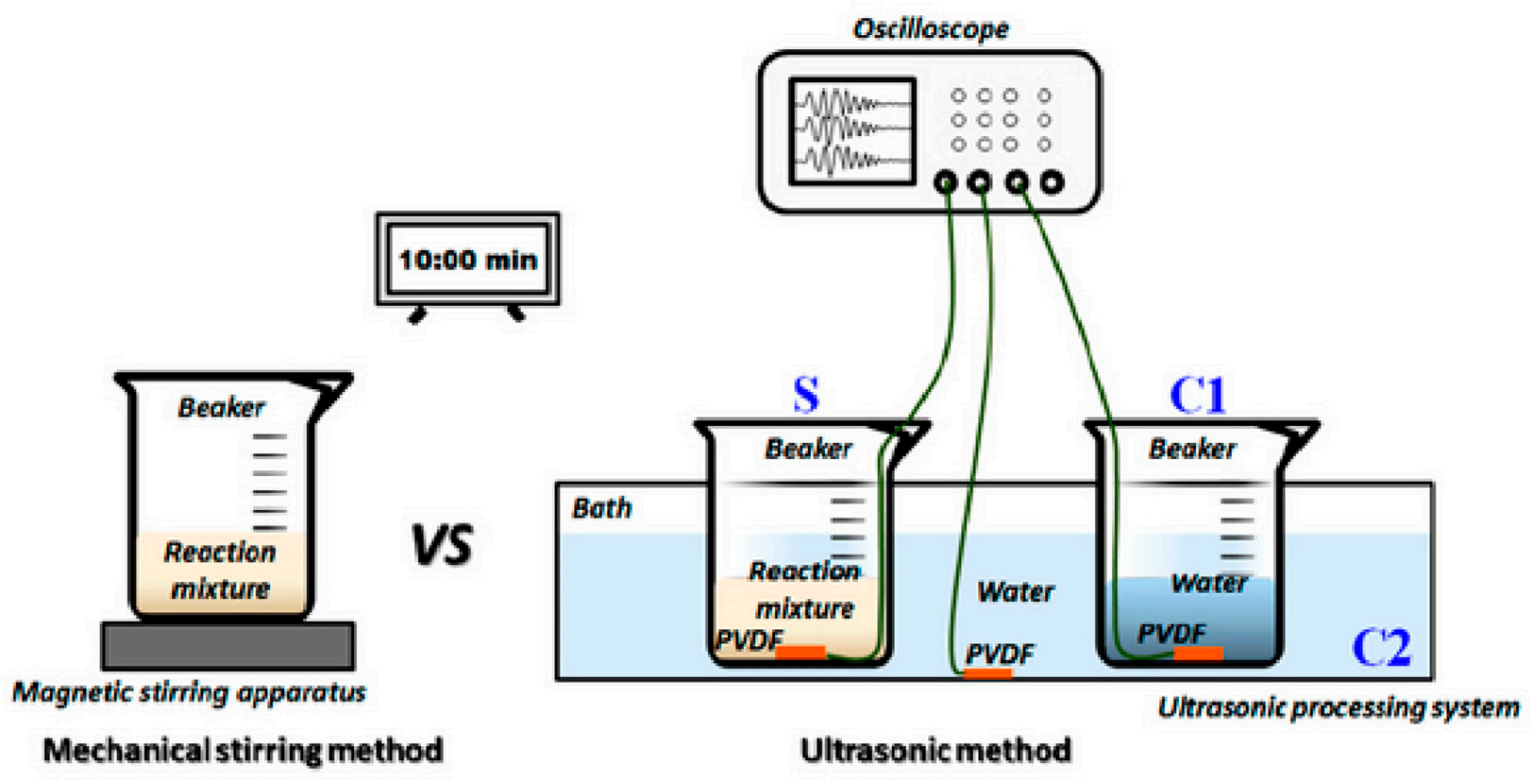
The diagram illustrates the process of monitoring ultrasonic fields while synthesizing nano-ZnO.

Ultrasonic irradiation has frequently been employed for generating nanoparticles during solution-based processes (Hajnorouzi et al., 2014). Using ultrasonic irradiation is the most effective and feasible method for producing pure materials that possess a variety of controlled features (El-Nahhal et al., 2017; Čech Barabaszová et al., 2019). In Zhang’s research, the physical process behind the synthesis of ZnO nanoparticles using ultrasound was revealed. The acoustic signal displayed four distinct stages of transformation initiated by ultrasound, which included fluctuations in the voltage due to the oscillation of cavitation bubbles, cycles of collapse, the highest level of voltage amplitude, and acoustic intensity (as shown in Figure 3) (Zhang L. et al., 2022).

#### Laser ablation

It is an effortless and dependable method for producing nanoparticles of virtually any type, such as semiconductors. Laser ablation synthesis has proven to be a successful approach for generating a wide range of nanoscale substances and offers the opportunity to examine a solid object within a liquid environment. This approach offers numerous advantages, such as the ability to regulate the ablation environment using inexpensive tools. In addition, by altering variables such as pulse laser length, laser frequency, pH levels of solution, heat, and surface-active agents, the size of the produced material can be changed (Farahani et al., 2016; Al-Nassar et al., 2019).

### A non-conventional method

#### Microfluidic reactor-based method

One possible approach to creating materials on a worktable is by using the microfluidic reactor-based method (Jin and Jin, 2019). Due to its distinctive qualities in generating nanoparticles through non-typical means and at smaller scales, this process presented numerous benefits. Furthermore, it was employed specifically for manufacturing ZnO nanoparticles. This method entails using few materials and results in targeted effects, conservation of the surroundings, rapid response speeds, minimal harm to the environment, and enhanced protection and health (Yu et al., 2018; McClements and Rao, 2011).

### Modifications

**FIGURE 4:**
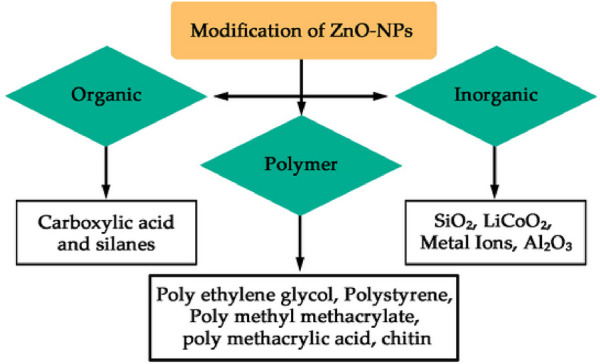
A diagram representing the commonly used techniques for modifying ZnO.

**FIGURE 5:**
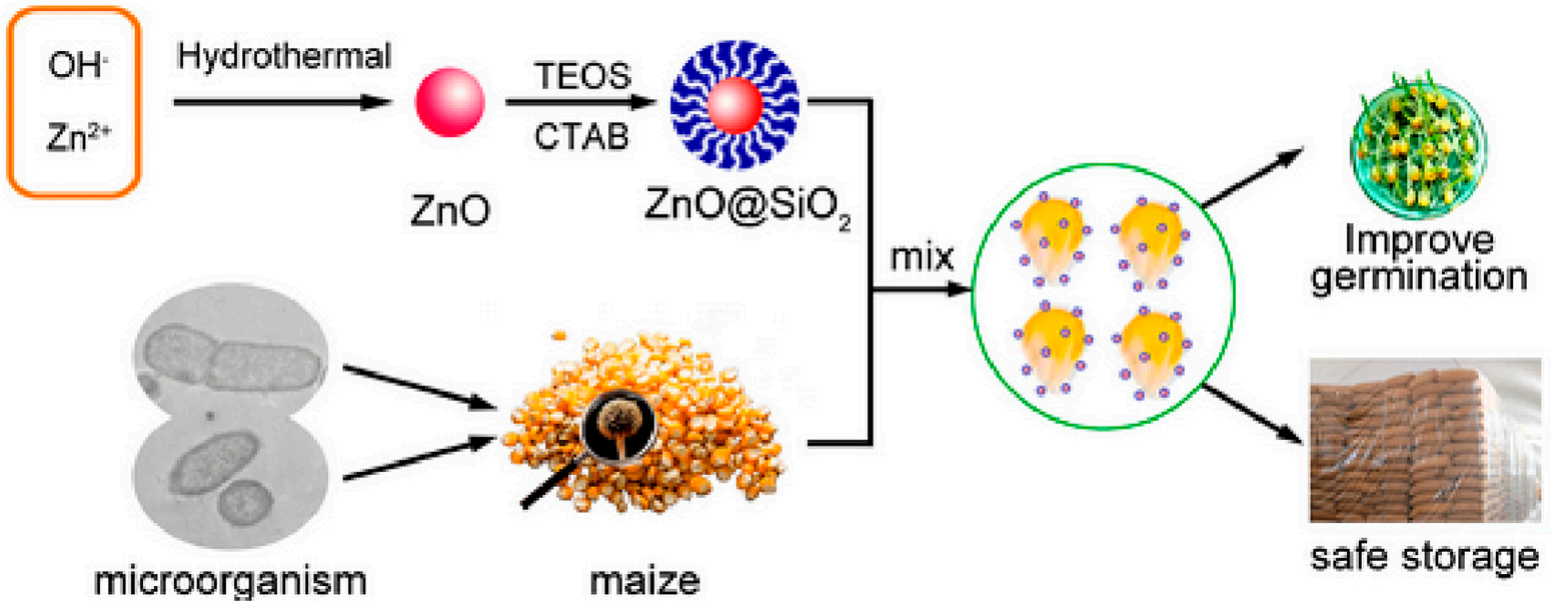
A diagram illustrating the process of creating and modifying ZnO, as well as its use in preventing the proliferation of bacteria and fungi on maize plants.

A product has the capacity to undergo changes in its size, composition, form, color, level of firmness, softness, and durability. The changes have the potential to improve the versatility of ZnO-NPs when they interact with different substances such as carboxylic acid, silanes, metal oxides, and polymers, as demonstrated in Figure 4. This has the potential to enhance the effectiveness of ZnO-NPs in terms of its lifespan, durability, and optical properties, ultimately making it more beneficial by increasing its advantages and significantly reducing any flaws (Chen et al., 2012; Wysokowski et al., 2015; Yung et al., 2017; Halbus et al., 2020). We here focus on a detailed collection of different methods for modifying ZnO-NPs that have been discussed in various sources. A growing body of research suggests that silica and trimethyl siloxane (TMS) have the ability to modify ZnO. The initial form of Zn carbonate hydroxide, known as ZCH, underwent heating to create the most refined ZnO particles. The method of precipitation was used to create zinc carbonate hydroxide (ZCH) using different materials such as ammonium hydroxide (NH_4_OH), ammonium bicarbonate (NH_4_HCO_3_), and zinc sulfate heptahydrate (ZnSO4⋅7H_2_O). During the processing of ZCH in water, the surface was modified *in situ* using hexamethyldisilazane (HMDS) and TEOS. In order to create extremely small ZnO particles, the ZCH was subjected to calcination and functionalization. This modification in the ZnO particles effectively resolved the problem of clumping together. The effectiveness of ZnO as a photocatalyst was diminished by the addition of silica, while the presence of an organic molecule enhanced its ability to form a harmonious connection with an organic matrix. To achieve the desired advantages, surface-modified ZnO was investigated as an exceptionally transparent material, capable of providing excellent shielding against UV rays, for the purpose of using these modifying agents. The methods and elements involved in the ZnO-NPs’ ability to combat yeast and algae were thoroughly examined (Gu et al., 2018; Artesani et al., 2020; Raha and Ahmaruzzaman, 2022). Scientists used TEOS and cetyltrimethylammonium bromide (CTAB) to devise a technique for producing a mesoporous silica coating on the ZnO-NPs, ultimately leading to the successful fabrication and examination of ZnO@mSiO2 nanoparticles. This technique effectively prevented direct contact between ZnO-NPs and maize, while still allowing use of their antibacterial properties. Moreover, this process may result in a reduction in ZnO-NP clustering and an enhancement of their dispersion, as shown in Figure 5 (Wojnarowicz et al., 2018b). Table 1 also provides a comprehensive overview about different methods of ZnO NP production.

**TABLE 1:** Different methods for production of ZnO NPs.

Method	Average crystalline size	Shape	Processing time	Primary solvent	Green principles/sustainable features	Reference
Hydrothermal method	33 ± 2 nm	Spherical	6.5 h	Double distilled water	Use of double distilled water minimizes contamination and ensures purity. Low temperature and pressure conditions reflect energy efficiency	Zhang et al. (2022a)
Precipitation method	18 nm	Granular	6 h (stirring) + centrifugation (2 min) + drying (6 h)	Deionized water and acetone	Use of deionized water minimizes contamination Low temperature and mild conditions reduce energy consumption Non-toxic acetone for washing. No harsh chemicals	Adam et al. (2018)
Chemical vapor transport method	Approx. 100 nm diameter for ZnO NWs	Nanowire and nanotube	Varied; up to several hours	Argon (carrier gas)	Use of high temperatures and controlled conditions to minimize waste	Güell et al. (2021)
Plant-mediated synthesis	16 nm	Spherical	7 h	Deionized water	Uses green chemistry principles with leaf extracts for nanoparticle synthesis and stabilization. Promotes environmental sustainability by potentially reducing chemical waste and energy usage during synthesis	Kamarajan et al. (2022)
Microorganism-mediated synthesis	∼46 nm (DLS)	Spherical/agglomerated	24 h (with 37°C incubation and multiple washing)	Deionized water (for Zn2+ suspension)	Biological synthesis using LAB strains, environmentally friendly, and no use of chemical reagents	Mohd Yusof et al. (2020)
Arc plasma	88–103 nm	Hexagonal column (with variations due to Ga-doping)	24 h (including various annealing and spraying steps)	Dry air and water (for dispersions)	Environmentally friendly due to minimal use of chemicals and uses dry air and water for dispersion	Islam et al. (2022)
Thermal evaporation	300–500 nm	Rods, spheres, nanowires, and films	1.5–2 h for growth	None (atmospheric air and no solvents used)	No harmful chemicals used, relies on high temperature and pressure, and reducing agents such as graphite, Si, Ge, Sn, and Pb enable reduction	Lv et al. (2010)
Physical vapor deposition	55–100 nm	Nanowires and fractal nanoferns	60 min (growth time at 450°C–600 °C)	None (atmospheric air and no solvents used)	The method relies on a physical vapor deposition process, utilizing minimal chemicals, and does not require toxic solvents. However, high temperatures are used, making it energy-intensive. It produces high-quality ZnO without the need for catalysts	Lyu et al. (2003)
Ultrasonic Irradiation	19.07 nm (average size)	Nanowires, regular, and uniform	10 h and 30 min	Ionic liquid ([Bmim] [BF4]), ethyl alcohol, and deionized water	Uses ionic liquid for better dispersion; the ultrasonic cavitation effect improves uniformity and reduces agglomeration, making the synthesis process efficient. However, requires energy for sonication and uses ionic liquids, which might raise environmental concerns	Zhang et al. (2022b)
Laser ablation	Not mentioned exactly	Spherical	15–20 min (including cleaning)	Distilled water with CTAB	CTAB prevents nanoparticle agglomeration and controls size; process requires significant energy and materials	Al-Nassar and Hussein (2019)
Microfluidic reactor-based method	Not mentioned exactly	Nanowires (ZnO)	∼1–2 min	Water (with BTEX mixture and CTAB for decontamination)	High efficiency in water decontamination, scalable for flow rates from 50 to 1000 μL/min; minimal dead volume	Azzouz et al. (2018)

### Zinc oxide nanoparticles and GI cancers

#### Zinc oxide nanoparticles and gastric cancer

Gastric cancer (GC) is accountable for a considerable number of fatalities caused by cancer on a global scale, ranking as the second most prevalent reason for mortality related to cancer (Marin et al., 2016). In later stages, chemotherapy is considered the most effective treatment for GC patients and commonly involves the use of oxaliplatin, 5-fluorouracil (5-FU), and cisplatin (DDP) as the main therapeutic agents (Ham et al., 2019; Zhai et al., 2019; Zheng et al., 2017). Despite improvements in the efficacy of chemotherapy, the overall survival rate is still deemed inadequate due to the persistence of chemotherapy resistance. This greatly hinders the effectiveness of GC therapies (Guo et al., 2019; Xu et al., 2018). The intricate and uncertain biochemical mechanisms that regulate the ability to withstand GC chemotherapy present a difficulty in fully comprehending them (Yuan et al., 2020). Therefore, immediate solutions for reducing the development of resistance to chemotherapy are highly necessary.

The process of autophagy involves the transfer of cellular components, such as malfunctioning organelles and extensive protein aggregates, to lysosomes for breakdown and recycling (Mizushima, 2007). Autophagy is crucial for maintaining a stable internal environment within cells and preventing harm caused by various stressors. In addition, it has an impact on both facilitating and impeding the development of cancer (Mathew et al., 2007). In specific circumstances, autophagy prompts the persistence of cancer cells by repairing their internal structures and increasing the generation of energy to fulfill their increased metabolic demands. In various circumstances, autophagy serves to impede cellular dysfunction and damage, thereby reducing the likelihood of tumor formation (Zhao et al., 2016). When autophagy is prevented, there is a marked connection to the resistance of GC cells to chemotherapy, and this resistance is diminished (Xi et al., 2019; YiRen et al., 2017). Autophagy is highly correlated with both cell differentiation and tumor development in GC (Caruso et al., 2018). Therefore, components related to autophagy may have the potential to act as dependable indicators of the outlook for advanced gastric cancer (Kim et al., 2019).

Miao and colleagues conducted a thorough investigation of how ZnO-NP impacts the resistance to chemotherapy in the progression of gastrointestinal cancer (Miao et al., 2021). The information they gathered indicated that ZnO-NPs effectively hindered the growth, movement, and infiltration, while also triggering apoptosis in GC cells. During this time, ZnO-NP exhibited a significant impact on reducing the half-maximal inhibitory concentration (IC50) of DDP to hinder the growth of DDP-resistant SGC7901/DDP cells. The levels of autophagy were elevated in DDP-resistant gastric cancer cells, as demonstrated by an increase in LC3II/LC3I and beclin-1 proteins and a decrease in p62 protein expression in SGC7901/DDP cells in comparison to SGC7901 cells. In terms of the mechanical aspect, ZnO-NP effectively inhibited the process of autophagy in GC cells. Nonetheless, administering DDP resulted in the activation of autophagy, although the addition of ZnO-NPs counteracted this response. In summary, *in vivo* experiments showed that ZnO-NPs effectively impeded the pace of tumor development in DDP-resistant gastric cancer cells. Researchers have found that ZnO-NPs have a significant impact on increasing the sensitivity of GC cells to chemotherapy by inhibiting the mechanism of autophagy. Their findings provide fresh insights into the mechanisms by which ZnO-NP influences GC cells’ resistance toward chemotherapy. The implementation of ZnO-NP could be a feasible choice for combating GC. It is crucial for future research to determine the clinical implications of ZnO-NP use in the treatment of GC (Miao et al., 2021).

*Morus nigra* (MN), commonly called black mulberry, is native to Southwestern Asia and has been grown in Europe and the Mediterranean area for numerous generations. The effects of MN on the body, both in terms of medicine and biology, have not been extensively studied compared to those of other *Morus* plant species. Extracts of MN containing several beneficial components have been used as herbal remedies for both humans and animals due to the ability to treat inflammation, reduce pigmentation, regulate blood sugar levels, lower cholesterol, combat obesity, fight against microbes, and potentially prevent cancer (Lim and Choi, 2019; Hussain et al., 2017; Budiman et al., 2018).

The main objective of Tang and colleagues was to generate MN-ZnO NPs through a range of analytical methods such as UV–Vis spectroscopy, TEM, SEM, FT-IR, EDX, and XRD, in order to evaluate their unique characteristics (Tang et al., 2020). Furthermore, the effectiveness of MN-ZnONPs in combating cancer in AGS cells was evaluated through the assessment of various elements, including the cell viability, alterations in the cell morphology suggestive of apoptosis through AO/EtBr staining, fluctuations in mitochondrial membrane potential, disruptions in cell division, levels of lipid peroxidation as measured by TBARS, amounts of antioxidant enzymes (SOD, GSH, and CAT), and the detection of reactive oxygen species. Moreover, the expressions of genes associated with cell death, specifically Bax, caspase-9, caspase-3, and Bcl-2, were measured using RT-PCR techniques. The scientists observed that the artificially produced MN-ZnONPs are circular, show clear crystalline characteristics, and possess a diverse range of functional groups. In addition, they observed that the use of MN-ZnONPs resulted in gastric cancer cell death. The MN-ZnONPs induced programmed cell death by stimulating the generation of reactive oxygen species and decreasing the mitochondrial membrane potential. These impacts were verified by observing the alterations in cell morphology, increased lipid impairment, decreased levels of antioxidants, and disruption of the cell’s reproductive process. Furthermore, to confirm the precise molecular mechanisms by which MN-ZnONPs induce cell death through altering gene expressions involved in apoptosis, the alterations in the levels of apoptotic indicators were meticulously recorded (Tang et al., 2020).

Different methods, including physical, chemical, and biological techniques, are commonly used to produce nanoparticles that possess diverse physical and chemical properties (Madhumitha et al., 2016). For example, several types of zinc salts have been employed in the hydrothermal or microwave-assisted synthesis methods for producing ZnO NPs, using Zn2+ ions as the primary building blocks (Baruah and Dutta, 2009; Hasanpoor et al., 2015). Unfortunately, the use of microwave-assisted synthesis methods is hindered by the high cost of the equipment and the absence of reaction monitoring, making them less accessible due to their complex procedures. The sol–gel approach is a popular and well-recognized method for producing ZnO NPs, frequently referenced in literature as a significant technique (Hasnidawani et al., 2016). This method has been acknowledged to face obstacles due to the expensive nature of the initial ingredients and challenges in accurately controlling the reaction conditions. The solution combustion-assisted technique is a significant method for creating ZnO NPs, which involves a self-sustaining reaction between a consistent solution, metal salts, and either organic or man-made fuel (Ranjithkumar et al., 2021). The use of this approach is primarily hindered by the requirements of ignition and the occurrence of reactions at temperatures ranging from moderate to high. The process of generating ZnO NPs using physical methods is affordable and has the added advantage of decreasing the radiation exposure and minimizing waste production. Furthermore, research has demonstrated that the implementation of methods involving living organisms leads to the production of almost fully decomposable nanoparticles. One disadvantage of using this approach is the inability to make changes to specific procedures (Parveen et al., 2016).

The concept of producing NPs using a flame-based method has gained considerable attention due to the various benefits it offers, such as being cost-effective, efficient, and easily accessible. This has resulted in its growing popularity as an alternative technique for NP manufacturing (Trommer et al., 2010). The initial discovery of this technique was credited to Ulrich, who subsequently applied it to create a significant number of nanoparticles (Ulrich, 1971; Strobel et al., 2006). The primary mechanism behind flame spray pyrolysis is the controlled release of metal ions from an aqueous solution, which are then transformed into a fine spray in a flame. This process ultimately results in the formation of small droplets as the solvent within the flame “burns” (Nunes et al., 2019). The process of pyrolysis converts the salt into a metal oxide, which then aggregates into clusters of NPs. These nanoparticles are gathered onto a surface or substrate (Nunes et al., 2019).

Du et al. used a financially viable method called flame spray pyrolysis to produce ZnO NPs. These particles were then studied for their specific ability to fight against cancer in the human stomach cancer cell line (AGS). The focus was also on understanding the internal cellular process of apoptosis that leads to their effectiveness (Du et al., 2023). The ZnO NPs that were produced underwent comprehensive characterization using multiple methods such as TEM, FT-IR, XRD, and DLS. Afterward, various levels of ZnO NPs were administered to both AGS and regular L-929 fibroblast cell lines, and the MTT assay was used to measure cell proliferation and determine the IC50 value for the NPs. Moreover, fluorometry and flow cytometry methodologies were employed to determine the levels of reactive oxygen species and programmed cell death. The scientists performed experiments to determine the levels of SOD and CAT activity in AGS cells, in order to evaluate the impact of ZnO NPs and oxidative stress on cellular damage. In order to assess the level of caspases-3/-8/-9 and the mitochondrial membrane potential (MMP), studies were carried out through experiments. To sum up, ZnO NPs were studied for their ability to activate the mitochondrial apoptosis signaling pathway in AGS cells through the evaluation of p53, Bax, Bcl-2, caspases-9/-8/-3, and cytochrome c levels using quantitative real time PCR and Western blot analyses. According to the results, ZnO NPs with a maximum absorption at 350 nm and an average size of 70 nm had a hydrodynamic radius of 92.89 nm and a zeta potential of −43.13 mV, and they effectively blocked the growth of AGS cells specifically compared to L-929 cells. This result was attained by a notable increase in the generation of ROS, initiation of programmed cell death, reduction in the activity of superoxide dismutase (SOD) and catalase (CAT), enlistment of caspases-9 and -3, and disturbance of the integrity of the mitochondrial membrane. Furthermore, the application of ZnO NPs led to an increase in the levels of p53, Bax, caspases-9/-3, and cytochrome c, while simultaneously reducing the expression of Bcl-2 at both the genetic and protein levels. Based on this study, ZnO NPs produced through flame spray pyrolysis have the potential to induce targeted anti-cancer properties on gastric cancer cells. This effect is achieved through the intrinsic apoptosis pathway (Du et al., 2023).

Curcumin, a vividly hued yellow seasoning, is a significant element of the enduring plant (turmeric), and it has been extensively researched as a chemopreventive agent. This compound is extracted from the *Curcuma longa* L. plant and is found in nature. This phenomenon has the capability to induce cellular demise, specifically in malignant cells, by causing harm to the DNA. Over the past 50 years, thorough studies have shown that curcumin has the ability to hinder the growth of various cancer cells in laboratory settings, as well as protect against cancer caused by carcinogens in rodents and slow down the development of tumors in animal models through xenotransplantation or orthotransplantation (Ji et al., 2012). Nanoparticles composed of ZnO vary in size and shape and possess distinct physical and chemical characteristics when compared to the larger, bulk version (Zhong Lin, 2004). One of the greatest benefits of incorporating ZnO into drug delivery systems is its low toxicity and stability in various environmental conditions. Furthermore, it has exhibited highly favorable results in biological settings (Hahn et al., 2012). The charge trapping states found in ZnO are primarily located on the surface, and they can be neutralized by applying inorganic coatings or by attaching organic molecules onto the surface of the ZnO NPs (Sandmann et al., 2015). ZnO NPs combined with drugs have the potential to enhance their ability to enter cancer cells by using hydrophobic and Coulombic interactions for intracellular delivery (Xiong, 2013; Nel et al., 2009). ZnO NPs have a stable state when the pH level is approximately 7, but they quickly break down when the pH drops below 6. Additionally, these nanoparticles are effective at drug delivery to their intended destination because tumor cells tend to remain stable at a pH level of 6. Luckily, ZnO is not harmful on its own, but once it breaks down, its Zn2+ ions can be toxic to cells. Since the intended destination had a lower acidity level (∼5.4), it is possible that this may have caused the nanocomposite to break down, activating the polymer connections and releasing the desired medication(s). At this stage, the ZnO nanocomposites dissolve and release the environmentally friendly Zn2+ ions. The sequence of actions involved in the medication administration process causes interruptions in cell metabolism, leading to the creation of reactive oxygen molecules, lipid damage, and DNA damage as a result (Ma et al., 2016).

### Zinc oxide nanoparticles and colorectal cancer

Colorectal cancer (CRC) was responsible for 1.93 million fresh cancer diagnoses in 2020, ranking it as the third most prevalent type of cancer following breast and lung cancer. Last year, it was the second leading cause for mortality, resulting in the death of 916,000 individuals, trailing closely behind lung cancer (Ferlay et al., 2019). Researchers and medical professionals face significant challenges when it comes to combating and treating colorectal cancer, a formidable and relentless disease. There has been an increase in the cases of colorectal cancer, especially among individuals in their early adulthood, underscoring the critical importance of implementing effective treatments and preventive measures (Verma et al., 2021). Although traditional treatments for cancer, like chemotherapy and radiation, have played a vital role in controlling the disease, they frequently have accompanying adverse reactions and are not always fully effective (Chen et al., 2016; Ceramella et al., 2022). Although there are numerous chemotherapy options for treating cancer, their effectiveness is limited by drug resistance and difficulty in distinguishing between healthy and cancerous cells, leading to incomplete results. Additionally, these treatments often require high doses of medication (Bayat Mokhtari et al., 2017). Historically, chemotherapy has been effective in destroying rapidly dividing cancer cells by disrupting their capacity to create DNA. The chemicals, without differentiation, have the potential to harm both healthy and cancerous tissues, resulting in undesirable side effects such as nausea and a decrease in weight. *Tinospora cordifolia,* also known as *T. cordifolia*, is a genetically diverse and sizable climbing shrub that is commonly used for medicinal purposes. It is deciduous and found in mountainous regions, producing greenish-yellow flowers. This particular plant is widespread in India and has numerous traditional medicinal advantages (Yates et al., 2022). However, a thorough examination of research results revealed that *T. cordifolia* plant extract has not been used to produce ZnO NPs before. Over the past two decades, significant efforts have been made to produce nanomaterials that are both environmentally friendly and cost-effective (Iqbal et al., 2018). Herbs are commonly used as the main source for NP production because they are readily available, easily accessible, and contain a large amount of potent reducing agents (Iqbal et al., 2018; Kamil Hussain et al., 2019).

Berehu et al. carried out an investigation where the methanol extract derived from the stem of *Tinospora cordifolia* was used in order to synthesize biogenic ZnO NPs. These NPs exhibited the potential to fight against colorectal cancer cells by using their anti-tumor characteristics (Berehu and Patnaik, 2024). The study utilized the production of biogenic ZnO-NPs by using a methanol extract derived from the stems of *Tinospora cordifolia* (resulting in ZnO-NPs TM), followed by assessing their effectiveness in combating HCT-116 cancer cells. The methods of UV–Visible spectrometry, FT-IR, XRD, SEM, and TEM were fully employed in order to examine and comprehend the biogenic ZnO NPs. In order to determine the success of using biogenically produced ZnO NPs as a cancer treatment, a range of assessments were performed. These included testing for cytotoxicity, staining with AO/EtBr and annexin V/propidium iodide (PI), measurement of mitochondrial membrane potential (MMP), analysis of ROS, and examining the activity of the caspase cascade. These evaluations were conducted to determine the effectiveness of biogenic ZnO-NPs as an anticancer agent. The IC50 values for HCT-116 and Caco-2 cells exposed to biogenic ZnO-NPs were 31.419 ± 0.682 μg/mL and 36.675 ± 0.916 μg/mL, respectively, representing the concentrations at which half of the cells were impacted. The results obtained through qRT-PCR testing showed a notable increase (with a p-value of less than or equal to 0.001) in the expressions of Bax and P53 mRNA in cells exposed to biogenic ZnO-NPs. The findings demonstrated that it hindered MMP activity and increased the generation of ROS. Similarly, our study carried out on live organisms demonstrated that naturally derived ZnO-NPs possess anti-tumor characteristics. Extensive studies on biologically derived ZnO nanoparticles, specifically those coated with TM, have proven to possess highly effective anti-cancer abilities. These results have been consistently replicated in controlled laboratory settings and animal trials, indicating the potential for using this as a feasible treatment method for cancer (Berehu and Patnaik, 2024).

For many centuries, *Swertia chirayita* has been an enduring plant species in the temperate Himalayan region. It has been naturally present there since ancient times. The Nepali neem plant is an enduring herb found in Nepal’s forests, often referred to as a yearly or 2-year species due to its consistent appearance (Aleem and Kabir, 2018). This specific plant, classified as either an herb or shrub, has a maximum height of 1.5 m and flourishes in the sub-temperate area of the Himalayan Mountains. *Swertia* species found in India have 40 varieties (Kumar and Van Staden, 2015). Renowned for its harsh flavor, *S. chirayita* has a lengthy history of being used in traditional medicine. This substance displays the capability to combat both Gram-positive and Gram-negative bacteria by hindering the growth of microorganisms. According to the documentation of Unani medicine, different parts of the plant are used to treat a range of health issues such as liver and heart problems, coughing, urinary problems, depression, swelling, nerve pain, and skin issues. It is frequently used as a supplement for digestion and a restorative agent for a range of stomach-related conditions, including dyspepsia, decreased appetite, and constipation. It has also proven to be helpful in addressing malaria, fever, and asthma (Khanal et al., 2014).

Ahmad and colleagues discovered that extracts from both crude and purified sources of *S. chirayita* had a considerable impact on inhibiting cell growth and activating apoptosis, serving as evidence of its effectiveness against cancer. *S. chirayita* is a vital component in various Ayurvedic treatments and has been included in the 1983 edition of the British Herbal Pharmacopoeia (Ahmad et al., 2021). Some of the main elements present in *S. chirayita* are secoiridoid glucosides, which include swertiamarin, sweroside, gentiopicroside, amarogentin, and amaroswerin. Additionally, there are several derivatives of tetrahydroxyxanthone (Wawrosch et al., 2005).

The researchers produced ZnO-NPs by combining the ethanolic and methanolic extracts of *S. chirayita* leaves. The resulting ZnO-NPs were then evaluated using techniques such as UV–Visible spectroscopy, FTIR, SEM, HRTEM, and XRD (Berehu and Patnaik, 2024). Researchers investigated the potential anticancer effects of the substance by conducting tests to measure its toxicity, including the MTT assay and acridine orange staining, as well as using quantitative real-time PCR on cells from both colorectal cancer (CRC) and non-cancerous cells (HEK-293). The diameter of the ZnO NPs formed through the use of the *S. chirayita* ethanolic extract is 24.67 nm, whereas those formed with the methanolic extract are circular have an average size of 22.95 nm. The MTT test revealed that the cancer cells (HCT-116 and Caco-2) were harmed by the nanoparticles, which was not the case for the non-treated cells (HEK-293). The IC50 levels for ZnO-NPs derived from ethanol and methanol were recorded as 34.356 ± 2.71 and 32.856 ± 2.99 μg/mL for HCT-116, 52.15 ± 8.23 and 63.1 ± 12.09 μg/mL for Caco-2, and 582.84 ± 5.26 and 615.35 ± 4.74 μg/mL for HEK-293. Acridine orange staining was employed to confirm that ZnO-NPs could induce apoptosis. Using qRT-PCR, it was determined that there was a significant increase in E-cadherin expression, along with a decrease in vimentin and CDK-1 expression levels. In summary, the results suggest that the synthesized ZnO-NPs may possess promising anti-cancer properties specifically against colorectal cancer (Berehu and Patnaik, 2024).

Researchers studied how ZnO-NPs and ANPs affect the HT29 colon cancer cell line in humans (Subramaniam et al., 2019). Zinc oxide and anthocyanin nanoparticles have a strong impact on suppressing the growth of HT29 colon cancer cells, but the level inhibition achieved varies according to the amount used. In addition, these miniscule particles have an impact on the electrical charge of cell membranes and hinder the development of cell clusters. The results suggest that ZnO NPs are more effective in diminishing the proliferation of colon cancer cells when compared to ANPs (Subramaniam et al., 2019).

The challenge in effectively using chemotherapy for treating CRC is a major hindrance, often resulting from a multitude of mechanisms occurring within cells. Certain elements that may contribute to an individual’s resistance to medication include an increase in the production of drug-carrying proteins, alterations in the affected components of the body, advancements in DNA repair mechanisms, and avoidance of predetermined cell demise. The primary markers linked to chemotherapy resistance in CRC include elevated levels of ABC transporters, alterations in the KRAS and BRAF genetic components, and modifications in the TP53 and APC genes. The role of these biomarkers is crucial in understanding and identifying resistance mechanisms, specifically for the purpose of targeting them for treatment strategies. Therefore, there is a demand for novel treatment methods that specifically focus on killing cancer cells while causing minimal harm. A possible choice could be oleuropein (OLP), a type of polyphenol present in olive leaf extract that has shown potential effectiveness in combating a range of cancers including those affecting the breast, liver, colon, thyroid, skin, and lungs (Tzekaki et al., 2021; Sherif and Al-Gayyar, 2018; Cárdeno et al., 2013; Giner et al., 2016; Bulotta et al., 2013; Preedy and Watson, 2020; Antognelli et al., 2019). According to a wealth of data, it has been noted that oleuropein demonstrates the capacity to reduce drug resistance in cancerous cells. Administering oleuropein results in an increase in p21, p53, and TNFRSF10B levels, while simultaneously causing a decline in Bcl-2 and Mcl1 levels in cisplatin-resistant ovarian tumors. Additionally, research has demonstrated that OLP is capable of effectively combating Tzb resistance in breast cancer cells as it works to reduce the levels of HER2 expression (Hashemi Sheikhshabani et al., 2021; Ruzzolini et al., 2018; Menendez et al., 2007). Previous studies have highlighted the potential of OLP to specifically target and disrupt pathways that contribute to the survival and proliferation of cancer cells in the context of CRC. This indicates that OLP is highly likely to be an effective solution for overcoming resistance to 5-FU in treatment. Park and colleagues synthesized a new compound called ZnO/Gold (Au)/OLP mesoporous silica nanoparticles (MSNs) that incorporates OLP and is applied onto multi-shell nanospheres made of zinc oxide and gold. These MSNs were subsequently examined for their efficacy in treating colon cancer cells that showed resistance toward 5-fluorouracil (Park et al., 2024). The ZnO/Au MSN composites primarily contained a combination of ZnO and gold ions, were circular, and did not result in any detrimental impact on cellular systems. The mixture of zinc oxide, gold, and oleic acid-modified mesoporous silica nanoparticles had a significant impact on decreasing both the survival rate of cells and the development of cancer stem cells, while also inducing programmed cell death in chemotherapy-resistant colorectal cancer cells. This was accomplished by the buildup of ROS and subsequent breaking down of DNA, resulting in cell death by necrosis. The combination of ZnO, Au, and OLP MSNs demonstrated the potential to enhance cell death via necrosis, ultimately exerting anti-tumor properties. The results showed that using ZnO/Au/OLP MSNs is an innovative approach for delivering 5-FU in the treatment of CRC (Park et al., 2024).

In their research, Alavi et al. investigated the impact of core-shell ZnO NPs (ZnO-NPs@polymer shell) combined with oxaliplatin (OXA) on colorectal cancer through various laboratory experiments and experiments on live mice (Alavi et al., 2023). In the last step of co-precipitation polymerization, ZnO nanoparticles were created. A coating made of a polymer was created on the surface of ZnO nanoparticles by harnessing the natural tendency of gadolinium (Gd) to bond with oxygen (III), leading to the production of ZnO–gadolinium NPs@polymer shell. The physical characteristics of the NPs were examined through multiple techniques including powder X-ray diffraction (PXRD), FT-IR, UV-Vis, FESEM, TEM, atomic force microscopy, dynamic light scattering, and z-potential. The antiproliferative effects of ZnO-Gd-OXA were measured using MTT. Furthermore, an investigation was carried out using a model of colon cancer in mice that involved implanting foreign tissue in order to assess its efficacy in impeding the growth and function of tumors. This research also evaluated the efficiency of the model through the use of histological staining methods, specifically H-E and trichrome staining, as well as gene expression analysis conducted with RT-PCR/ELISA. Furthermore, a biochemical assessment was performed to quantify the amounts of malondialdehyde, thiols, superoxide dismutase, and catalase. The PXRD examination established that ZnO NPs were successfully produced and had a crystallite size measuring 16.8 nm. The findings from EDX analysis indicated that the ZnO NPs had a flat shape and included platinum. The examination using TEM showed that the particles were circular with a diameter of 33 ± 8.5 nm. However, the size measurements in a fluid environment suggested that the particles were heavily aggregated. The results of the research indicated that ZnO-Gd-OXA demonstrated a successful ability to impede the growth of tumors by stimulating the production of reactive oxygen species and hindering fibrosis. This sheds light on the potential efficacy of ZnO-Gd-OXA as a viable treatment alternative for colorectal cancer and emphasizes the need for further research and studies (Alavi et al., 2023).

### Zinc oxide nanoparticles and pancreatic cancer

One must consider the degree of tissue penetration as a significant aspect when addressing tumors situated deep in the body, such as those found in the pancreas. Pancreatic cancer, specifically the most common form called pancreatic ductal adenocarcinoma (PDAC), presents significant difficulties in treatment due to its distinctive growth and inherent ability to resist usual methods of cancer treatment (Sarantis et al., 2020; Zeng et al., 2019; Conte and Cauda, 2022). When it comes to medical ailments, therapies such as photodynamic therapy (PDT) have demonstrated effectiveness in specifically targeting the difficult goal of reducing tumor size. Regrettably, the location of the pancreas in the human body does not allow for effective stimulation with light. A large variety of different types of remote triggers have been proposed. A prime example of this idea can be illustrated by using repetitive force pulses, explicitly through ultrasound (US) or shock waves (SWs), which are abrupt and potent disruptions containing both compressive and tensile waves. These waves cause a sudden and notable change in the pressure and density of a substance or material (Wan et al., 2016; Canavese et al., 2018). These fluctuations result in an abrupt and remarkable alteration in the force and compactness of a substance or element. In addition, at lower radiation levels, ultrasound remains a safe choice for healthy tissues. On the other hand, higher energy levels can be used to destroy cancer cells through methods like tumor thermoablation, which involve focused ultrasound procedures (Stewart et al., 2003). Numerous molecules have been recommended as compounds that promote this phenomenon and are referred to as sonosensitizers. These are devices that can generate heat or trigger the generation of reactive oxygen species (ROS) in response to ultrasound, creating a targeted treatment for the specific area (Rosenthal et al., 2004). From this perspective, non-particle solutes such as gold, titanium oxide, and zinc oxide have been suggested to successfully enhance ultrasound treatment and have shown great potential in this field (Sazgarnia et al., 2011; Brazzale et al., 2016; Wang F. et al., 2022; Vighetto et al., 2021; Matos et al., 2020; Dumontel et al., 2022; Vighetto et al., 2019; Racca et al., 2020). The vast body of literature has extensively debated the superiority of nanoparticles over organic sonosensitizer molecules. However, this is still a topic of debate (Li et al., 2021). NPs have a greater capacity to disperse in water-based environments in biological contexts than organic compounds, making them highly suitable for use in living beings. Nanoparticles may be present within the cancerous tissue by either taking advantage of the enhanced permeation and retention effect or by using specialized biomolecules on their surface that specifically aim for cancer cells (Costley et al., 2015).

In their study, Carofiglio et al. aimed to employ Fe:ZnO NPs as a versatile tool for both treatment and detection purposes. They also aimed to determine the potential of these nanoparticles to be more efficient against pancreatic ductal adenocarcinoma when used in combination with shock waves, a type of mechanical pressure (Carofiglio et al., 2022). Iron-ZnO NPs are generated through the use of oleic acid as a capping agent and subsequently adjusted with amino-propyl groups. In their preliminary discoveries, researchers record the exceptional characteristics of the pure ZnO nanoparticles, specifically in relation to their magnetic traits, stability in solution, capability to interact with cells, and proficiency in infiltrating BxPC-3 pancreatic cancer cells in a controlled environment. These nanoparticles containing iron and zinc oxides are also harmless for use in conjunction with fully functioning cells in the pancreas. Following this, they perform a cell treatment that involves the use of both shock waves and Fe:ZnO NPs once the particles have been incorporated into the cells. They also examine how NPs contribute to fostering collaborative effects in the extracellular region. The results suggest that using both NPs and shock waves independently does not have a negative impact on cells. However, when merged together, they result in a heightened frequency of cellular death within 24 h. Several procedures are envisaged afterward, which involve the breakdown of tiny particles, the creation of unattached molecules, and the disruption or entry into the cell membrane. Studies have confirmed that when iron is present in ZnO NPs, they are capable of degrading inside cells and releasing zinc ions. Moreover, the use of shock waves has the potential to cause cells to break open by inducing membrane permeability. Alternatively, in this situation, the production of reactive oxygen species has been ruled out. Cell death, whether through apoptosis or necrosis, is evident. This preliminary research illustrates the capability for potential future application in treating tumors that are deeply embedded, specifically in the case of pancreatic cancer, which continues to be a major and pressing issue in the medical field, resulting in a substantial number of deaths (Carofiglio et al., 2022).

Zhao and his team investigated the eco-friendly method of producing ZnO NPs using *Anacardium occidentale* leaf extract through a sustainable synthesis approach (Zhao et al., 2018). ZnO NPs were generated by boiling a combination of 10 mL of *A. occidentale* leaf extract and 30 mL of 0.1 M zinc nitrate (ZnNO3) at 60°C for a duration of 3 h. The nanoparticles were thoroughly examined using spectroscopic and microscopic techniques, including FT-IR, TEM, XRD, energy-dispersive EDS, and UV–Vis. The hexagonal pattern of the ZnO nanoparticles was discovered through the use of X-ray diffraction analysis. TEM examination confirmed the nanoparticle structure. Additionally, the scientists measured the toxicity levels of the created nanoparticles on cells of human pancreatic cancer. The results of the cytotoxicity experiments have confirmed that the produced ZnO nanoparticles exhibit different levels of damage to pancreatic cancer cells, with the strength becoming more pronounced as the concentrations increase (Zhao et al., 2018).

### Zinc oxide nanoparticles and hepatocellular carcinoma

A current research project examined the impact of ZnONPs on red blood cells (RBCs) and determined that the absorption and ability to cause blood cell breakdown of these particles were dependent on their size. The findings indicated that FA has the ability to alleviate the adverse effects on RBCs (Preedia Babu et al., 2017). FA, a form of phenolic compound found extensively in plant cell walls, has displayed numerous advantageous properties such as being an antioxidant, fighting against cancer and inflammation, and safeguarding the liver and nervous system (Kumar and Pruthi, 2014; Zduńska et al., 2018).

Ezhuthupurakkal and their team conducted a comprehensive examination of ferulic acid-conjugated ZnO NPs (ZnONPs-FAC) through a multifaceted approach involving computational, spectroscopic, and microscopic techniques (Ezhuthupurakkal et al., 2018). In a controlled laboratory environment, the possibility of having anticancer properties has been explored by evaluating cell viability and morphology, generation of reactive oxygen species, mitochondrial membrane permeability, DNA damage using comet assay, and identification of distinct biomarkers like 8-OHdG, Ki67, and γ-H2AX. Furthermore, the impact of the therapy on the cell cycle was researched through examination, as well as through Western blot analysis. Moreover, the potential for anticancer effects on a living being was assessed by analyzing tissue specimens through histopathological and immunohistochemical methods following the induction of liver cancer with DEN. The results clearly showed that using ZnONPs-FAC can induce apoptosis and hinder the growth of DEN-induced HCC. The study documents the healing properties that can be attained by using a combination of nanoparticles and phytochemicals, suggesting a new method of using combined chemotherapy (Ezhuthupurakkal et al., 2018).

In their research, Abbasi et al. strived to produce environmentally friendly ZnO NPs by using different components of *Silybum marianum* (L.) Gaernt., such as seeds, naturally growing plants, laboratory grown plantlets, and cultures of callus cells. Subsequently, they performed comprehensive examinations and evaluated the biological impact of the NPs (Abbasi BH. et al., 2019). The ZnO nanoparticles were extensively scrutinized and studied by employing techniques like XRD, FTIR, and SEM. The ability of the newly formed nanoparticles to withstand temperature changes was further evaluated through thermo-gravimetric analysis. Researchers successfully created durable crystalline nanoparticles, ranging from 30.8 to 46.0 nm, using different tissues from *S. marianum*. These naturally occurring substances have shown a wide range of possible applications in the realm of biology, such as shielding against oxidation, hindering the activity of α-amylase, possessing antibacterial qualities, and displaying harmful effects on cells. The use of seed extract to create ZnO nanoparticles proved to have the strongest antibacterial impact (measuring 20 ± 0.98 mm) against *S. aureus* (ATCC-6538). Using seed extract to create ZnO nanoparticles demonstrated the most potent antioxidant properties, showing levels of 27.7 ± 0.9 µgAAE/mg, 23.8 ± 0.7 µgAAE/mg, and 12.7% ± 1.9% for total antioxidant capacity (TAC), total reducing power (TRP), and the DPPH-free radical scavenging assay (FRSA) respectively. The HepG2 cells were shown to be affected by the toxic properties of all the man-made ZnO nanoparticles. Interestingly, the ZnO nanoparticles exhibited a remarkable aptitude for effectively interacting with living beings, as evidenced by the outcomes of toxicity and hemolysis assessment using brine shrimp and human red blood cells. The effectiveness of using seed extract in producing NPs was found to be the most beneficial among all other types of NPs that were developed and utilized, making it a promising option for future applications (Abbasi B. H. et al., 2019).

*Punica granatum* L., commonly known as the pomegranate fruit, is a member of the Punicaceae botanical family and is recognized for its excellent nutritional properties, thanks to its abundant supply of phenolic compounds and gallic acid (GA) (Elfalleh et al., 2012). Based on the results, pomegranate juices possess a significantly elevated number of antioxidants, surpassing that possessed by green tea and red wine by nearly threefold (Shaban et al., 2013). GA possesses multiple hydroxyl groups and is found in a variety of sources such as pomegranates, fruits, plants, and food items. There is evidence that it possesses diverse capabilities, such as combating viruses and bacteria, hindering the production of melanin, and safeguarding against cancer in various cell types (Dorniani et al., 2012; Ashrafizadeh et al., 2021).

Unfortunately, gallic acid’s potential in the pharmaceutical industry is limited due to its low solubility, poor absorption by the body, and susceptibility to changes in stability. In order to enhance the effectiveness of administering targeted medications and enhance the body’s capacity to process and use GA, it was transformed into nanoparticles (Elfalleh et al., 2012).

For a study, GA was chosen for reduction and coating, leading to the creation of zinc–gallic acid nanoparticles (Zn-GANPs) (AboZaid et al., 2024). The newly formed tiny particles were subsequently investigated in a live rat model of liver cancer. Exposure to DEN led to a rise in levels of inflammatory indicators including AFP and NF-κB p65, liver enzymes such as AST and ALT, γ-GT, globulin, and total bilirubin, while also causing a decrease in protein and albumin levels. The research demonstrated an elevation in MDA concentrations and oxidative damage to liver cells, along with a surge in Bcl-2 expression. In contrast, the presence of Zn-GANPs resulted in notable decreases in caspase-3 levels and those of crucial antioxidants like GSH and CAT. This led to a significant reduction in levels of lipid peroxidases, AST, ALT, and γ-GT. Additionally, Zn-GANPs demonstrated a considerable ability to increase the levels of CAT and GSH (p < 0.05). Moreover, Zn-GANPs caused cell cycle arrest at the S and G2/M stages, ultimately leading to the occurrence of G0/G1 apoptosis. These results were correlated with higher amounts of caspase-3 and lower amounts of Bcl-2 and TGF-β1. The employment of zinc-coated graphene nanoparticles showed significant enhancements and reversal of liver histology and ultrastructure in rats that were induced with DEN. According to the data, Zn-GANPs have the ability to impede and treat liver injury and cancer resulting from DEN (AboZaid et al., 2024).

Bashandy and colleagues investigated ZnO-NPs on hepatocellular carcinoma in rats. They administered 5 and 10 mg/kg ZnO-NPs daily for 8 weeks and found that the treatment of these animals with these NPs resulted in the inhibition of cancer biomarkers including glypican-3 (GPC3), VEGF, and alpha-fetoprotein (AFP). Moreover, improvement of histopathology and biomarkers of the liver along with the improvement in glucose metabolism and lipid profile are other effects of this NP (Bashandy et al., 2021).

### A discussion on the mechanisms involved in ZNO-NP-induced cancer inhibition, current limitations, and future directions

Zinc oxide nanoparticles (ZnO-NPs) have gained significant attention in cancer research due to their various biological effects, including their potential therapeutic role in gastrointestinal (GI) cancers, such as gastric, colorectal, and hepatocellular carcinoma. Below is a mechanistic discussion of how ZnO-NPs affect GI cancer, focusing on their anticancer properties, molecular mechanisms, and the pathways involved. ZnO-NPs affect GI cancer cells through various pathways that lead to cell death, inhibition of proliferation, and suppression of tumor growth. ZnO-NPs can generate reactive oxygen species (ROS) upon entering the cancer cells. ROS are highly reactive molecules that can induce oxidative stress, leading to cellular damage, DNA fragmentation, and ultimately apoptosis (programmed cell death) (Akhtar et al., 2012; Simon et al., 2000). The overproduction of ROS is a critical mechanism by which ZnO-NPs trigger apoptosis in cancer cells (Akhtar et al., 2012). By increasing ROS levels, ZnO-NPs disrupt the cellular redox balance, resulting in mitochondrial damage, activation of apoptotic pathways, and inhibition of cancer cell survival (Kou et al., 2019; Wu et al., 2022). Expressions of key antioxidant enzymes such as superoxide dismutase (SOD), catalase (CAT), and glutathione (GSH) are downregulated in response to ZnO-NPs, contributing to the accumulation of ROS and oxidative damage (Bashandy et al., 2021; El-Shorbagy et al., 2019). This mechanism is particularly relevant in GI cancers where the high metabolic demands of tumor cells make them more susceptible to oxidative damage (Akhtar et al., 2012). ZnO-NPs have been shown to activate intrinsic apoptosis pathways in GI cancer cells. ZnO-NPs have been shown to increase the expression of p53, a tumor suppressor protein that regulates cell cycle arrest and apoptosis in response to DNA damage (Yang et al., 2021; Setyawati et al., 2013). p53 activation can lead to cell cycle arrest at the G1/S checkpoint and subsequent apoptosis (Anjum et al., 2021; Padmanabhan et al., 2019). ZnO-NPs alter the expressions of pro-apoptotic (Bax) and anti-apoptotic (Bcl-2) proteins. Increased Bax/Bcl-2 ratio favors mitochondrial outer membrane permeabilization, leading to cytochrome c release and activation of caspases (Akhtar et al., 2012; Efati et al., 2023). ZnO-NPs trigger the activation of caspases, especially caspase-3, caspase-9, and caspase-8, which are critical for executing apoptosis by cleaving various cellular substrates involved in survival (Pei et al., 2023; L et al., 2020). These apoptotic signaling events lead to the breakdown of cell integrity, release of pro-apoptotic factors, and activation of cell death pathways. Additionally, ZnO-NPs reduce the mitochondrial membrane potential (MMP), further enhancing apoptosis in cancer cells (L et al., 2020; Patrón-Romero et al., 2022). ZnO-NPs inhibit cancer cell proliferation in several ways. They interfere with cell cycle progression by affecting critical regulatory proteins (Rahman and Mustafa, 2023; Setyawati et al., 2015). For example, ZnO-NPs have been shown to reduce the levels of cyclins (e.g., cyclin D1, cyclin E) and cyclin-dependent kinases (CDKs), which are crucial for the transition between different phases of the cell cycle. This results in cell cycle arrest at the G1/S or G2/M checkpoints, thereby preventing cancer cell division and proliferation (Wang et al., 2023; Vallabani et al., 2019). In addition to directly inducing cancer cell death, ZnO-NPs can inhibit angiogenesis, the process by which new blood vessels form to supply nutrients and oxygen to tumors. Tumors require a constant supply of oxygen and nutrients, and ZnO-NPs have been shown to reduce the expressions of angiogenesis-related markers such as VEGF (vascular endothelial growth factor). This suppression of angiogenesis limits the tumor’s ability to grow and metastasize (Tada-Oikawa et al., 2015; Barui et al., 2017). ZnO-NPs also exhibit anti-metastatic properties. They can inhibit the migration and invasion of cancer cells by disrupting the signaling pathways that regulate cell adhesion, migration, and cytoskeletal dynamics. ZnO-NPs affect the expression of matrix metalloproteinases (MMPs) and integrins, which are crucial for the degradation of extracellular matrix components and cell movement (Srivastav et al., 2019; Mittag et al., 2022). ZnO-NPs affect autophagy in GI cancer cells, which can either enhance or inhibit their response to chemotherapy. In some cases, autophagy supports cancer cell survival, especially under conditions of metabolic stress or during chemotherapy. ZnO-NPs can induce autophagic processes by upregulating autophagy-related proteins such as LC3II, beclin-1, and p62 (Zhou et al., 2022; Bai et al., 2017; Roy et al., 2014). However, in the context of cancer therapy, the inhibition of autophagy by ZnO-NPs can reduce the survival of chemotherapy-resistant cancer cells (Martinez-Outschoorn et al., 2010; Liu et al., 2013). ZnO-NPs inhibit the autophagic process by reducing the levels of autophagy-related proteins, thereby sensitizing cancer cells to chemotherapy and evading cell death (Yang et al., 2020). ZnO-NPs can induce DNA damage in cancer cells through the generation of ROS. The ROS cause oxidative damage to DNA, which can result in DNA strand breaks, base modifications, and mutations, leading to cell cycle arrest and apoptosis (Condello et al., 2016; Zijno et al., 2015). ZnO-NPs have the potential to be used in combination with traditional chemotherapy agents (e.g., cisplatin, oxaliplatin, and 5-FU) to enhance their therapeutic efficacy (Wu et al., 2022; Racca et al., 2023). ZnO-NPs sensitize cancer cells to chemotherapy by reducing the IC50 values of chemotherapeutic agents, thus making the cancer cells more susceptible to drug-induced cell death (Othman et al., 2022; Pairoj et al., 2021). This combination therapy is particularly beneficial for overcoming chemotherapy resistance, which is a major limitation in GI cancer treatment (Racca et al., 2023; Pairoj et al., 2021).

Although zinc oxide nanoparticles (ZnO-NPs) have demonstrated significant potential in treating GI cancers in preclinical models, several limitations must be addressed before these nanoparticles can be translated into effective clinical treatments. One of the major drawbacks in the current research on ZnO-NPs for GI cancer treatment is the limited number of *in vivo* and human clinical trials. Most of the available studies are conducted on cell lines or animal models, which do not fully replicate the complex human physiology and tumor microenvironment. *In vitro* and animal studies provide valuable insights into the anticancer activity of ZnO-NPs, but their bioavailability, distribution, toxicity, and efficacy in humans are not well-understood (Raha and Ahmaruzzaman, 2022; Oprea et al., 2014). ZnO nanoparticles should be non-toxic to normal tissues and exhibit biocompatibility (Naser et al., 2023; Oleshko et al., 2020). For gastrointestinal tract applications, this means they must not cause adverse immune responses, inflammation, or other harmful effects upon systemic administration or localized application in the gastrointestinal tract as some studies suggested the hyperproliferation or malignant transformation of cells after the exposure with ZnO NPs (Meng et al., 2020; Meng et al., 2022). The bio-distribution and accumulation of ZnO-NPs in tumors remain unclear. Nanoparticles can accumulate in organs such as the liver, spleen, and kidneys, which might cause off-target effects or toxicity (Zhu et al., 2022; Chen et al., 2023). Furthermore, tissue penetration is another challenge, especially for solid tumors like those found in the GI tract (Barua and Mitragotri, 2014). Although nanoparticles are beneficial in overcoming biological barriers, their ability to reach deep tissues, like pancreatic or colorectal tumors, remains limited (Barua and Mitragotri, 2014). To improve specificity and minimize off-target effects, ZnO nanoparticles need to be functionalized with targeting molecules (such as antibodies, peptides, or small molecules) that bind specifically to biomarkers or receptors overexpressed in GIT tumor cells (e.g., EGFR and VEGF). This enhances the selectivity of ZnO nanoparticles for tumor cells, sparing healthy tissues (Pei et al., 2024; Yang and Guo, 2024). ZnO nanoparticles must be designed to take advantage of penetration into tumor cells for efficient tumor targeting. Additionally, their surface charge and hydrophilicity can influence their ability to penetrate and be retained within the tumor (Bisht and Rayamajhi, 2016). Moreover, most studies focusing on ZnO-NPs use them in skin or breast cancer treatments, but data on their effectiveness and safety in GI cancers are lacking (Chelladurai et al., 2021; Mohammed et al., 2019). The size, shape, and surface properties of ZnO-NPs play a significant role in their efficacy and toxicity. However, the synthesis of ZnO-NPs with a consistent size and shape remains challenging. The uncontrolled growth of nanoparticles can lead to formation of aggregates or heterogeneous particle distributions, which affect their pharmacokinetics and therapeutic efficacy (Raha and Ahmaruzzaman, 2022; Nair et al., 2009). Although surface modification with biomolecules can improve targeting and reduce toxicity, the uniformity and stability of these modifications still need improvement (Jung et al., 2020). Without precise control over the surface charge and coating, the interactions with biological systems can be unpredictable (Jung et al., 2020). Although ZnO-NPs are generally considered safe at low concentrations, high doses or prolonged exposure can lead to cytotoxicity and inflammation in healthy tissues (Ryu et al., 2014; Jin et al., 2021). The release of Zn2+ ions from ZnO-NPs in an acidic environment (like the tumor microenvironment) could lead to oxidative stress and damage to normal cells, potentially causing side effects (Plum et al., 2010). Despite the detailed mechanistic studies conducted *in vitro* and in animal models, the precise molecular mechanisms through which ZnO-NPs exert their anticancer effects in humans are not fully understood. Understanding the molecular interactions between ZnO-NPs and human cancer cells is crucial for optimizing their use in clinical settings. In particular, mechanisms such as ROS generation, apoptosis induction, autophagy inhibition, and immune modulation need to be further elucidated in human cancer models. Despite the challenges outlined above, ZnO-NPs hold great promise in cancer therapy. To move forward, several future research directions are crucial to ensure that ZnO-NPs can be effectively translated into clinical treatments for GI cancer. The most immediate need is to conduct more *in vivo* studies in animal models that more closely resemble human physiology and tumor behavior. These studies should focus on long-term toxicity, pharmacokinetics, and tumor-targeting capabilities of ZnO-NPs. Researchers should also explore the use of animal models that mimic human GI cancers, particularly those involving the pancreas, stomach, and colon, to better understand the behavior of ZnO-NPs in deep tissues. Additionally, human clinical trials should be initiated, beginning with Phase I trials to assess the safety, dosage, and side effects of ZnO-NPs in humans. Success in early-phase trials would enable progression to Phase II and III trials, focusing on the efficacy and comparative performance against current chemotherapy treatments. Future research should focus on improving the synthesis methods to achieve homogeneous size distribution and precise surface functionalization. This could involve using biomolecules or polymeric coatings to enhance the specificity and reduce the toxicity of ZnO-NPs. Surface modifications with targeting ligands that recognize specific cancer cell receptors (such as those overexpressed in GI tumors) could improve the tumor selectivity and reduce off-target effects. Biomolecules such as proteins, peptides, antibodies, or aptamers can be conjugated to the surface of ZnO NPs to enhance the targeting specificity. These biomolecules are chosen for their ability to selectively bind to receptors or antigens overexpressed on the surface of cancer cells, facilitating precise delivery of ZnO NPs to tumor sites (Corso et al., 2008; Taratula et al., 2009; Wang, 2009). A promising future research direction is to use ZnO-NPs in combination with other cancer therapies. Combination therapies have the potential to overcome the limitations of individual treatments. For example, ZnO-NPs could be combined with chemotherapy, radiotherapy, or immunotherapy to enhance the overall therapeutic effect. ZnO-NPs may help sensitize tumors to chemotherapy drugs (such as 5-FU, cisplatin, or oxaliplatin) by reducing drug resistance mechanisms, as shown in the studies with DDP-resistant gastric cancer cells. Furthermore, nanoparticle-based drug delivery systems could be developed, where ZnO-NPs act as carriers for chemotherapeutic agents or targeted therapies, improving the bioavailability and precision delivery of drugs to cancer cells. Recent studies suggest that ZnO-NPs may play a role in modulating the immune response. Exploring how ZnO-NPs affect immune cells, such as macrophages, T-cells, and dendritic cells, could open new avenues for combining ZnO-NPs with immunotherapy strategies. ZnO-NPs might enhance the immune system’s ability to recognize and attack cancer cells, potentially improving immune checkpoint inhibitor therapies (Saptarshi et al., 2015; Roy et al., 2015). Before ZnO-NPs can be widely used in human therapies, their long-term toxicity must be fully assessed. Research should focus on biodegradable ZnO-NPs that break down safely in the body, minimizing the risk of cumulative toxicity. The biocompatibility and clearance of ZnO-NPs need to be optimized, particularly concerning the accumulation of nanoparticles in vital organs and tissues.

## Conclusion

In conclusion, zinc oxide nanoparticles (ZnO-NPs) hold significant promise as a therapeutic agent for gastrointestinal (GI) cancers, including gastric, colorectal, and pancreatic cancers. Their ability to induce reactive oxygen species (ROS) production, disrupt mitochondrial integrity, activate apoptosis, and inhibit cancer cell proliferation presents an exciting avenue for cancer treatment. ZnO-NPs’ capacity to target specific tumor cells, sensitize them to chemotherapy, and reduce metastasis makes them a powerful addition to cancer therapy. Moreover, their use in combination with other therapeutic modalities, such as chemotherapy, radiotherapy, or immunotherapy, holds potential for overcoming treatment resistance and improving the efficacy.

However, the clinical translation of ZnO-NPs faces challenges, including limited *in vivo* and human studies, concerns about toxicity, and the need for precise control over their size, shape, and surface functionalization. Long-term safety studies and human clinical trials are essential to fully understand the pharmacokinetics, bio-distribution, and potential adverse effects of ZnO-NPs in cancer patients. Additionally, further research into their mechanisms of action, such as immune modulation and autophagy regulation, will be crucial in optimizing their therapeutic application. With continued innovation and research, ZnO-NPs could have the potential to become a valuable tool in the fight against GI cancers.
